# Systematic review and REMARK scoring of renal cell carcinoma prognostic circulating biomarker manuscripts

**DOI:** 10.1371/journal.pone.0222359

**Published:** 2019-10-22

**Authors:** Marco A. J. Iafolla, Sarah Picardo, Kyaw Aung, Aaron R. Hansen

**Affiliations:** 1 Division of Medical Oncology and Hematology, Princess Margaret Cancer Centre, University Health Network, Toronto, Ontario, Canada; 2 University of Toronto, Toronto, Ontario, Canada; 3 Livestrong Cancer Institute and Dell Medical School, University of Texas at Austin, Austin, Texas, United States of America; University of Mississippi Medical Center, UNITED STATES

## Abstract

**Background:**

No validated molecular biomarkers exist to help guide prognosis of renal cell carcinoma (RCC) patients. We seek to evaluate the quality of published prognostic circulating RCC biomarker manuscripts using the Reporting Recommendations for Tumor Marker Prognostic Studies (REMARK) guidelines.

**Methods:**

The phrase “(renal cell carcinoma OR renal cancer OR kidney cancer OR kidney carcinoma) AND circulating AND (biomarkers OR cell free DNA OR tumor DNA OR methylated cell free DNA OR methylated tumor DNA)” was searched in Embase, Medline and PubMed March 2018. Relevant manuscripts were scored using 48 REMARK sub-criteria for a maximal score of 20 points.

**Results:**

The search identified 535 publications: 33 were manuscripts of primary research and were analyzed. The mean REMARK score was 10.6 (range 6.42–14.2). All manuscripts stated their biomarker, study objectives and method of case selection. The lowest scoring criteria: time lapse between storage of blood/serum and marker assay (n = 2) and lack of flow diagram (n = 2). REMARK scores were significantly higher in publications stating adherence to REMARK guidelines (p = 0.0307) and reporting statistically significant results (p = 0.0318).

**Conclusions:**

Most RCC prognostic biomarker manuscripts poorly adhere to the REMARK guidelines. Better designed studies and appropriate reporting are required to address this urgent unmet need.

## Background

Renal cell cancers (RCC) are pathologically diverse with variable outcomes for patients with similar stage disease and treatment. Clinicopathological and radiological features comprise the primary RCC prognostic parameters. Despite these efforts, the natural history of RCC can still be unpredictable: small tumors < 4cm can harbor metastatic disease at time of diagnosis [1] and upwards of 40% of patients with lymph node metastases at time of nephrectomy may still be alive at 5 years post-resection [2]. While the International Metastatic RCC Database Consortium risk score can help predict prognosis and treatment response [3], there still remains a need to characterize disease states more precisely and better select management strategies. Currently no molecular biomarker has been validated for prognosis or treatment prediction, and no such marker has been integrated into routine clinical care for patients with RCC. Therefore, new biomarkers are urgently needed to improve prognostic accuracy that may inform clinical trial design, treatment selection and surveillance guidelines.

At least 16 RCC prognostic models exist that incorporate various clinical, laboratory and radiographic features, with each model examining discrete populations of patients (e.g. post-nephrectomy, prior vascular endothelial growth factor [VEGF] inhibitors, failed immunotherapy, etc.) [4]. However, few models have been validated and none are applicable to a broad group of RCC patients. New efforts to prognosticate RCC patients have focused on gene expression profiles from primary tumors, and results have suggested a possible association with overall survival in both RCC [5, 6] and other tumor types [7–9]. Most promising to date are loss-of-function mutations in the PBRM1 gene showing clinical benefit in an independent validation cohort of metastatic RCC patients treated with inhibitors of programmed cell death protein-1 or its ligand [10]. Further, the PBRM1 mutations can also help predict response and prognosis to VEGF inhibitors: RCC patients treated on the COMPARZ trial harboring PBRM1 mutations correlated with better overall survival (OS), progression free survival (PFS), objective response rate and enhanced the angiogenic microenvironment [11]. Despite these results, the analyses were performed on tumor samples that necessitate either invasive biopsy or archival specimens that may have insufficient tumor content or inadequate quality, and both modalities are subject to tumor heterogeneity.

One strategy undergoing investigation to address these limitations is the development of minimally-invasive blood-based biomarkers. Yet despite a wide range of cellular and molecular features being studied in RCC for over 15 years, there still remains a paucity of validated circulating prognostic biomarkers. This is not unique to RCC: less than 1% of promising oncologic biomarkers become clinically useful [12]. To help improve the transparency and quality of prognostic biomarker studies, the National Cancer Institute and the European Organisation for Research and Treatment of Cancer created the REporting recommendations for tumour MARKer prognostic studies (REMARK) in 2005 [13]. A goal of REMARK was to provide a methodological and reporting framework for investigators developing prognostic biomarkers in order to ensure that biomarkers were appropriately and accurately tested, and increase the likelihood of validation or corroboration in subsequent confirmatory studies. Presumably, failure to develop and validate a biomarker may arise from poor study design, methodological differences, non-standardized assays that lack reproducibility, statistical analyses with either misleading results or based on small sample sizes that lack the ability to create meaningful conclusions, or because the marker analyzed does not have prognostic impact. The challenge is to ensure that biomarkers are properly tested, do not fail or succeed because of flawed methodologies, and are appropriately reported to the scientific community.

Our study hypothesized the paucity of validated circulating RCC biomarkers is secondary to inadequate methodology and reporting, which could be demonstrated by failure of adherence to the REMARK guidelines. Hence, we performed a systematic review of the literature to determine the number of primary research manuscripts investigating RCC circulating prognostic biomarkers, and subsequently scored each valid manuscript using the REMARK criteria. The objective of our study was to review the quality of design and reporting in studies investigating prognostic circulating biomarkers in patients with RCC.

## Methods

### Literature search and publication organization

This literature search was guided by the Preferred Reporting Items for Systematic Review and Meta-analysis (PRISMA) statement [14]. The S1 File lists the PRISMA checklist. The following terms were used to search PubMed (March 23, 2018), Medline (March 29, 2018) and Embase (March 29, 2018) without any limit on date of past publications: “(renal cell carcinoma OR renal cancer OR kidney cancer OR kidney carcinoma) AND circulating AND (biomarkers OR cell free DNA OR tumor DNA OR methylated cell free DNA OR methylated tumor DNA).” Please see S2 File for the full rationale and MeSH terms included. We defined inclusion and exclusion criteria *a priori*: inclusion criteria consisted of manuscripts analyzing RCC prognostic circulating blood-based biomarkers; exclusion criteria were duplicates, non-RCC papers, or RCC studies limited to case reports, review papers, or abstract publications. Search results were exported into CSV file format for review. All publications were independently reviewed and organized by two authors (MI and SP) into one of three possible categories: publications examining RCC circulating prognostic biomarkers, publications not examining RCC circulating prognostic biomarkers, and publications that were unclear about examining RCC circulating prognostic biomarkers. Any persistent disagreements in organization between authors MI and SP were adjudicated by author AH. Among the publications examining RCC circulating prognostic biomarkers, only primary research manuscripts were subjected to REMARK scoring; reviewed papers and abstract-only publications were not amendable to REMARK appraisal. Valid manuscripts were then sub-classified based upon their investigated biomarker; categories were created if ≥ 2 publications were analyzing the same biomarker, and papers analyzing a biomarker not examined in another publication were classified as “other”. Publications that were not suitable or of unclear significance were also sub-classified. Attempts to clarify publications of unclear significance were assessed by contacting the corresponding author or, in the case of unclear abstracts, subsequent publications were searched to determine if the abstract pertained to RCC prognostic circulating biomarkers.

### REMARK scoring and prognostic parameters

The REMARK criteria consists of a checklist of 20 items [13], and each item can be further divided into multiple sub-categories [15]. To ensure a consistent interpretation and application of the REMARK criteria, all authors examined the REMARK sub-criteria in tandem and selected those of highest yield: each RCC prognostic biomarker manuscript was evaluated according to 48 separate sub-criteria for a maximum score of 20 points. A full list of the criteria and point per criteria is listed in Table 1. Authors MI and SP independently scored all relevant manuscripts and any disagreements were reviewed by author AH. Further, the following variables were also collected: specific prognostic metrics (e.g. OS, PFS, cancer-specific survival and recurrence free survival or disease free survival), stating adherence to REMARK guidelines, location of study, year of publication, sample size studied, histology of RCC investigated, stage of RCC investigated, statistically significant results reported and reporting of hazard ratios.

**Table 1:** The 48 sub-criteria used to score the valid RCC circulating prognostic biomarker publications and the number of publications awarded each point.

REMARK criteria number	Criteria number used in score	Criteria description *	Potential points awarded	Papers meeting criteria (max 33)
**Introduction**				
1	1	Marker stated.	0.33	33
	2	Objective stated.	0.33	33
	3	Pre-specified hypothesis stated.	0.33	3
**Materials and Methods**				
2	*Patients*			
	4	Source of patients.	0.33^‡^	24
	5	Inclusion criteria (i.e. stage of cancer).	0.33^‡^	21
	6	Exclusion criteria.	0.33^‡^	12
	7	If applicable: how specific cases were included if drawn from a parent study.	0.25^§^	7
3	8	Details of treatment.	0.5	20
	9	Timing of therapy relative to specimen collection.	0.5	25
4	*Specimen characteristics*			
	10	Methods of preservation.	0.33^‡^	21
	11	Storage.	0.33^‡^	23
	12	Time between time of storage and time of marker assay.	0.33^‡^	2
	13	If applicable: if controls are used, then details on the control’s morbidities, medications, sex, age, etc.	0.25^§^	0
5	*Assay methods*			
	14	Amount of specimen required to perform the assay.	0.33^‡^	11
	15	Strategies employed to reduce the measurement error.	0.33^‡^	9.5^b^
	16	Blinding of the person making the marker assessment to clinical outcomes.	0.33^‡^	3
	17	If applicable: multicenter studies must state if single reviewers or reference laboratories are used to reduce variability in marker measurements.	0.25^§^	3
6	*Study design*			
	18	Time period cases were taken.	0.25^¶^	27
	19	The end of follow-up period.	0.25^¶^	5
	20	Median follow-up time.	0.25^¶^	17
	21	Marker measurements were extracted retrospectively from existing records, assays were newly performed using stored specimens, or assays were performed in real time using prospectively collected specimens.	0.25^¶^	33
	22	If applicable: patients were stratified by clinicopathologic factors.	0.20^a^	0
7	23	The endpoint should be defined precisely.	1	13.5^b^
8	24	Fully define all variables.	1	14
9	25	Either sample size calculation, or effect size calculation given the pre-determined sample size.	1	5
10	*Statistical analysis methods*			
	26	Describe statistical methods with sufficient detail for verification.	0.5	32
	27	Must state that “all data was accounted for” or “no missing data occurred”.	0.5^b^	6.5
11	28	For continuous variables: clarify whether the data were kept on the original scale or log transformed, and indicate whether the relationship was modeled as linear or non-linear. For categorized variables: specify the cutpoints and how they were chosen.	1	32
**Results**				
12	*Data*			
	29	The study must show either a flow diagram (e.g. CONSORT), or a study profile diagram.	0.5	2
	30	Report the number of patients and the number of events.	0.5	18.5^b^
13	31	Distributions of basic demographic variables and standard prognostic variables.	0.5	29.5^b^
	32	Description of the distribution of the marker of interest.	0.5	9
14	*Analysis and presentation*			
	33	The association of the tumor marker with standard prognostic variables.	1	15.5^b^
15	34	Univariable relation between a categorical marker and outcome.	0.33	24
	35	Univariable confidence intervals.	0.33	16
	36	Univariable P-value.	0.33	29
16	37	Multivariable relation between a categorical marker and outcome.	0.33	23
	38	Multivariable confidence intervals.	0.33	19
	39	Multivariable P-value.	0.33	22
17	40	The study must evaluate whether the new marker maintains some association with clinical outcome after accounting for these standard prognostic variables.	0.25	8
	41	Confidence intervals.	0.25	4
	42	P-value.	0.25	5
	43	Discussion and explanation of how these standard variables have been selected.	0.25	4
18	44	Must test their model with one of the following: test their assumption, sensitivity analysis, or internal validation analyses or external validation studies.	1	6
**Discussion**				
19	45	One of the following: acknowledgment of any biases or inconsistencies in the data, limitations of the assay methods, or limitations of the design or data analysis methods.	0.5	27
	46	Comment on whether the results are consistent with, or differ from, the general tendency in previous studies and offer potential explanations for differences.	0.5	29
20	47	A discussion if the biomarker is clinically useful.	0.5	30
	48	Future research plans.	0.5	27

* Adapted from the 2005 REMARK publication [16] and the subsequent 2012 expanded REMARK Explanation and Elaboration edition [15]

^‡^ = each criteria was 0.25 points if criteria

^§^ is relevant to the publication

^¶^ = each criteria was 0.2 points if criteria

^a^ is relevant to the publication

^b^ = these criteria divided in two in the event only partial reporting occurred

### Statistical analysis

The REMARK scores were summarized using descriptive statistics such as mean and range. Pearson’s correlation was used for continuous variable correlation with REMARK score: journal impact factor, year of publication and sample size studied were analyzed. Impact factors were determined on October 9, 2018 by searching either InCites Journal Citation Reports [17] or other sources [18–23] in the event the journal was not available on InCites. Year of publication was based on the following hierarchy depending on information available: the year the manuscript was accepted for publication, year published online, and then year of periodical publication. Student’s paired t-test (for 2 categories) or ANOVA (for > 2 categories) were used to compare categorical variable correlation with REMARK score: 1) statement of adherence to REMARK guidelines, 2) location of study, 3) histology of RCC included, 4) stage of RCC included, 5) report of statistically significant results, and 6) type of survival metric that met statistical significance.

## Results

### Literature search results

The search identified 252 Embase results, 146 Medline results and 394 PubMed results. The PubMed results had 98 and 146 overlapping results with Embase and Medline, respectively. Embase had 13 repeated publications within its own Embase search: either the abstract was repeated twice (n = 5), or the abstract was repeated but under a different Unique Identifier (twice n = 5; three times n = 1). Note that one abstract had its Unique Identifier repeated twice and had the abstract repeated under a different Unique Identifier.

**Fig 1:**
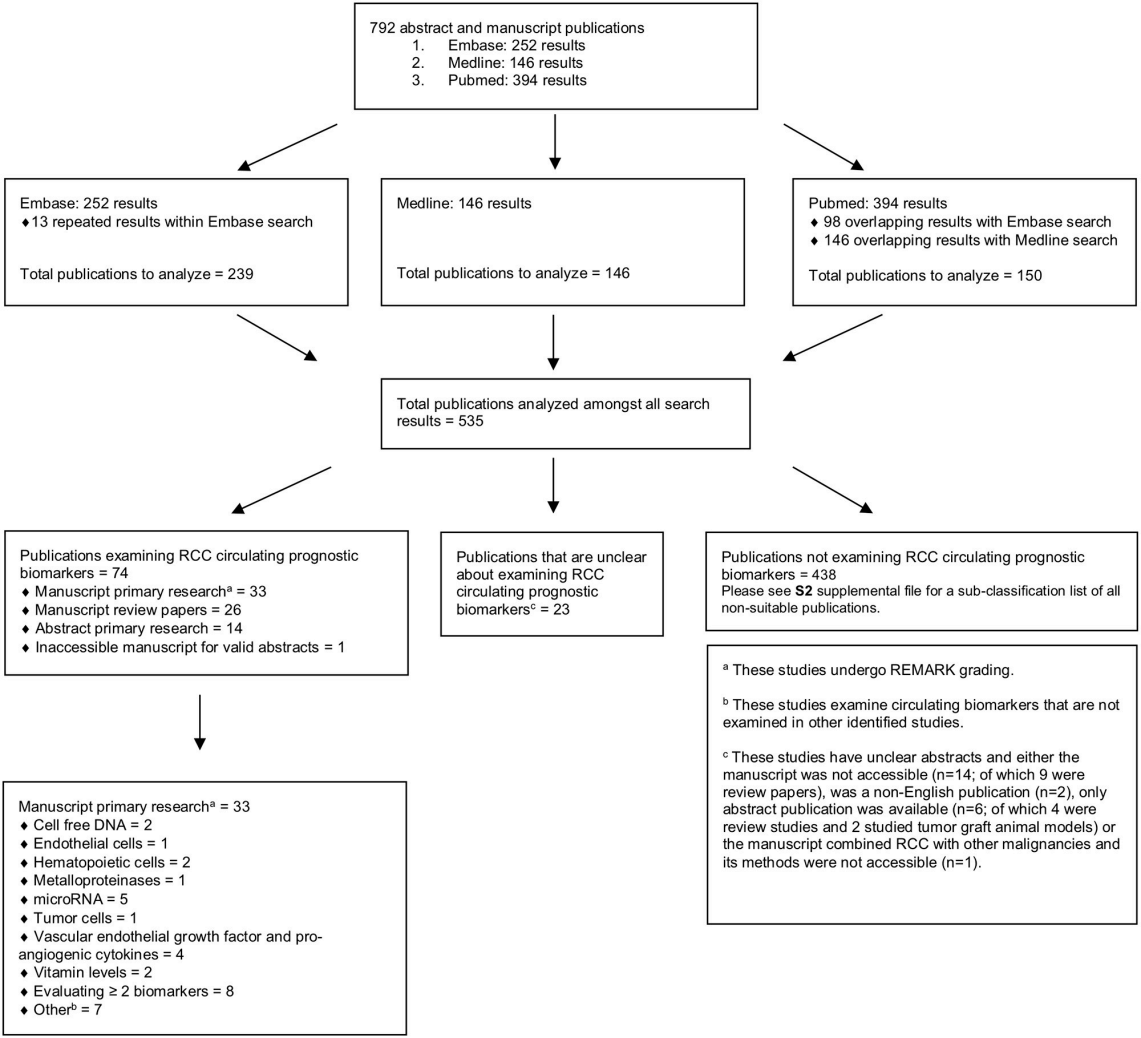
CONSORT flow diagram. Summary of results from the literature search and sub-classification of publications into one of three categories: publications examining RCC circulating prognostic biomarkers, publications not examining RCC circulating prognostic biomarkers, and publications that are unclear if examining RCC circulating prognostic biomarkers.

In total, 535 unique publications were identified: 74 examined RCC prognostic circulating biomarkers, 438 did not examine RCC prognostic circulating biomarkers, and 23 were unclear if they examined RCC prognostic circulating biomarkers. Among the RCC prognostic circulating biomarkers publications, 33 were manuscripts of primary research and were organized into 10 different categories; manuscripts evaluating ≥ 2 biomarkers (n = 8) and those meeting our definition of “other” (i.e. papers analyzing a biomarker not examined in other publications) (n = 7) comprised the majority. Although excluded from our final analysis, 26 review papers and 14 abstracts reported on RCC circulating prognostic biomarkers. One manuscript with a valid abstract was unable to be obtained despite attempts to contact the corresponding author. Publications not examining RCC prognostic circulating biomarkers were organized into 53 different categories (see S3 File): publications not reporting on diagnostic, predictive or prognostic biomarkers (n = 129) and publications examining RCC diagnostic circulating biomarkers (n = 46) were the largest categories. Publications of unclear significance were due to unclear abstracts, of which inaccessible manuscripts (n = 14) and abstract-only publication (n = 6) were the largest criteria composing this category. Among the inaccessible manuscripts, nine pertained to review articles and no additional attempts were made to access the manuscript for additional investigation. The five remaining abstracts were unable to be obtained despite attempts to contact the corresponding authors. One manuscript of unclear significance commented on the prognostic potential of circulating tumor DNA, but combined RCC with other malignancies and its methods were inaccessible, making the results uninterpretable for the purpose of this study. Among the unclear abstracts, four abstracts pertained to review studies and two studied tumor grafts in animal models. One additional abstract was initially listed as unclear, but was subsequently published and revealed to only report on predictive circulating biomarkers (this was categorized into the section “did not examine RCC prognostic circulating biomarkers”). Fig 1 summarizes these results in a CONSORT diagram [24]. Although categories were created if ≥ 2 publications were analyzing the same biomarker, the categories “endothelial cells”, “metalloproteinases” and “tumor cells” only have one manuscript present due to the same biomarker undergoing evaluation in a manuscript examining more than one biomarker.

### Valid manuscript descriptive statistics

**Fig 2:**
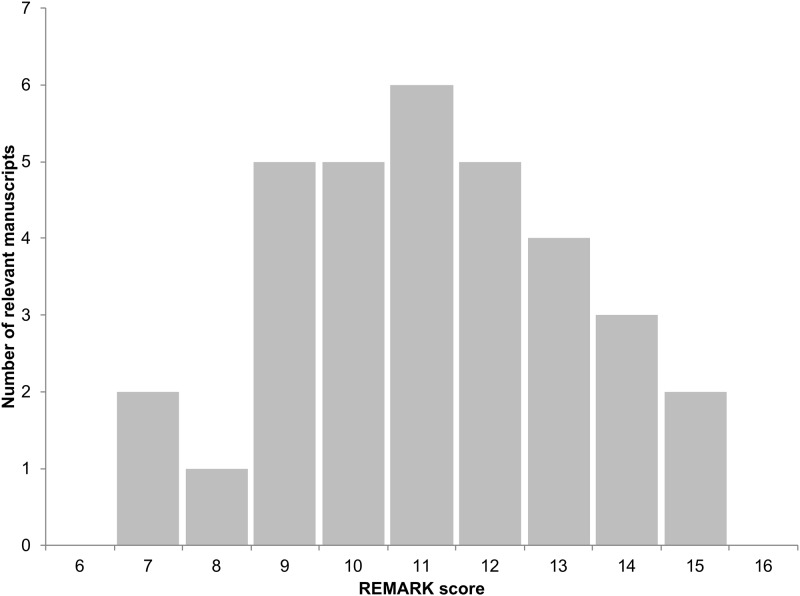
Distribution of REMARK scores in valid manuscripts. Histogram depicting the range of REMARK scores from relevant RCC circulating prognostic biomarker manuscripts identified in this study.

Thirty three manuscripts were included for review. As displayed in the Fig 2 histogram, the mean REMARK score was 10.6 (range 6.42–14.2; maximum score was 20). All manuscripts stated their marker, study objectives and method of case selection. The lowest scoring criteria were: description of time between storage of blood/serum and marker assay (n = 2); flow or study profile diagram (n = 2); blinding of the person making the marker assessment to clinical outcomes (n = 3); and pre-specified hypotheses (n = 3). In total, 20 (42%) of the REMARK sub-criteria were addressed in < 50% of the manuscripts. The Results section of the REMARK division was the least frequently reported with only a median 15 (46%) manuscripts satisfying these sub-criteria, relative to the Introduction, Methods and Discussion having a manuscript median number of 33 (100%), 17 (52%) and 28 (85%), respectively. The Assay Methods and Data REMARK sub-divisions were the least adhered to with a manuscript median number of 9.5 (29%) and 13.75 (42%), respectively, addressing these sub-criteria. Only three studies acknowledged an attempt to adhere to the REMARK criteria. Table 1 summarizes the number of publications addressed the 48 sub-criteria, and S1 Table lists all publications analyzing circulating RCC biomarkers and the REMARK score for the manuscripts.

The majority of the studies were conducted in Europe (n = 19; 58%), examined mixed RCC histologies (n = 20; 61%) and had mixed RCC TNM staging (n = 17; 52%). In total, 30 publications (91%) reported a statistically significant association between their circulating biomarker and a prognostic outcome, of which OS was the most common outcome reported (n = 15; 46%). The mean impact factor was 5.85 with a large range (1.2–13.926). The mean year of publication was 2012 (range 2004–2018). There was a large variation in RCC patient sample size (mean 188, range 7–750), and 18 manuscripts (55%) utilized RCC sample sizes < 100 patients. Table 2 summarizes additional descriptive statistics of this review. In total, 27 manuscripts (82%) reported hazard ratios. S2 Table lists all valid manuscripts with their corresponding REMARK sub-criteria points and correlative variables used in the statistical analysis.

**Table 2:** Descriptive statistics of pertinent variables from RCC circulating prognostic biomarkers publications and their continuous and categorical variable statistical analysis with REMARK scores.

TABLE 2. Descriptive statistics				
*Description*		*Mean (range)*	*n (%)*	*p-value*
REMARK score		10.60 (6.417–14.17)	-	-
Impact factor		5.85 (1.2–13.926)	-	-
Year of publication		2012 (2004–2018)	-	-
Sample size		188 (7–750)	-	-
*Description*		*Mean REMARK score (range)*	*n (%)*	*p-value*
Stating conformity to REMARK criteria	Yes	13.03 (11.67–13.58)	3 (9.1)	0.0307
	No	10.36 (6.41–14.17)	30 (91)	
Location of study	Asia	10.52 (6.42–13.17)	5 (15)	0.7429
	Europe	10.41 (6.92–14.17)	19 (58)	
	North America	11.06 (9.17–13.33)	9 (27)	
Histology	Clear-cell only	11.03 (6.92–14.17)	13 (39)	0.3448
	Mixed histology	10.33 (6.42–13.58)	20 (61)	
RCC stage investigated	After curative intent^a^	N/A	1 (3)	0.431
	Metastatic or locally advanced	10.75 (7.83–13.33)	15 (46)	
	Mixed staging	10.32(6.42–14.17)	17 (52)	
Statistically significant results	Statistically significant results	10.84 (6.92–14.17)	30 (91)	0.0318
	No statistically significant results	8.19 (6.42–9.12)	3 (9.1)	
	Univariate significance only	10.02 (7.83–13.33)	8 (24)	0.1938
	Multivariate significance only	10.08 (8.25–12.83)	4 (12)	
	Both univariate and multivariate significance^b^	11.38 (6.92–14.17)	18 (55)	
Survival metric with statistical significance	Significance to 1 survival metric	10.55 (6.92–14.17)	21 (64)	0.2134
	Significance to > 1 survival metric	11.54 (8.83–13.33)	9 (27)	
	Overall survival^c^	11.40 (8.25–14.08)	15 (46)	0.102
	Progression free survival^c^	11.11 (7.83–13.33)	13 (39)	
	Cancer specific survival^c^	10.43 (6.92–14.17)	9 (27)	
	Recurrence free survival^c^	10.88 (8.08–13.17)	4 (12)	
	N/A	8.19 (6.42–9.17)	3 (9.1)	

^a^ = curative intent and mixed groupings were combined due to curative intent n = 1

^b^ = includes papers that also report a mix of univariate and multivariate significance to different survival metrics

^c^ = includes papers that also report significance to > 1 survival metric

### Associations with REMARK score

**Fig 3:**
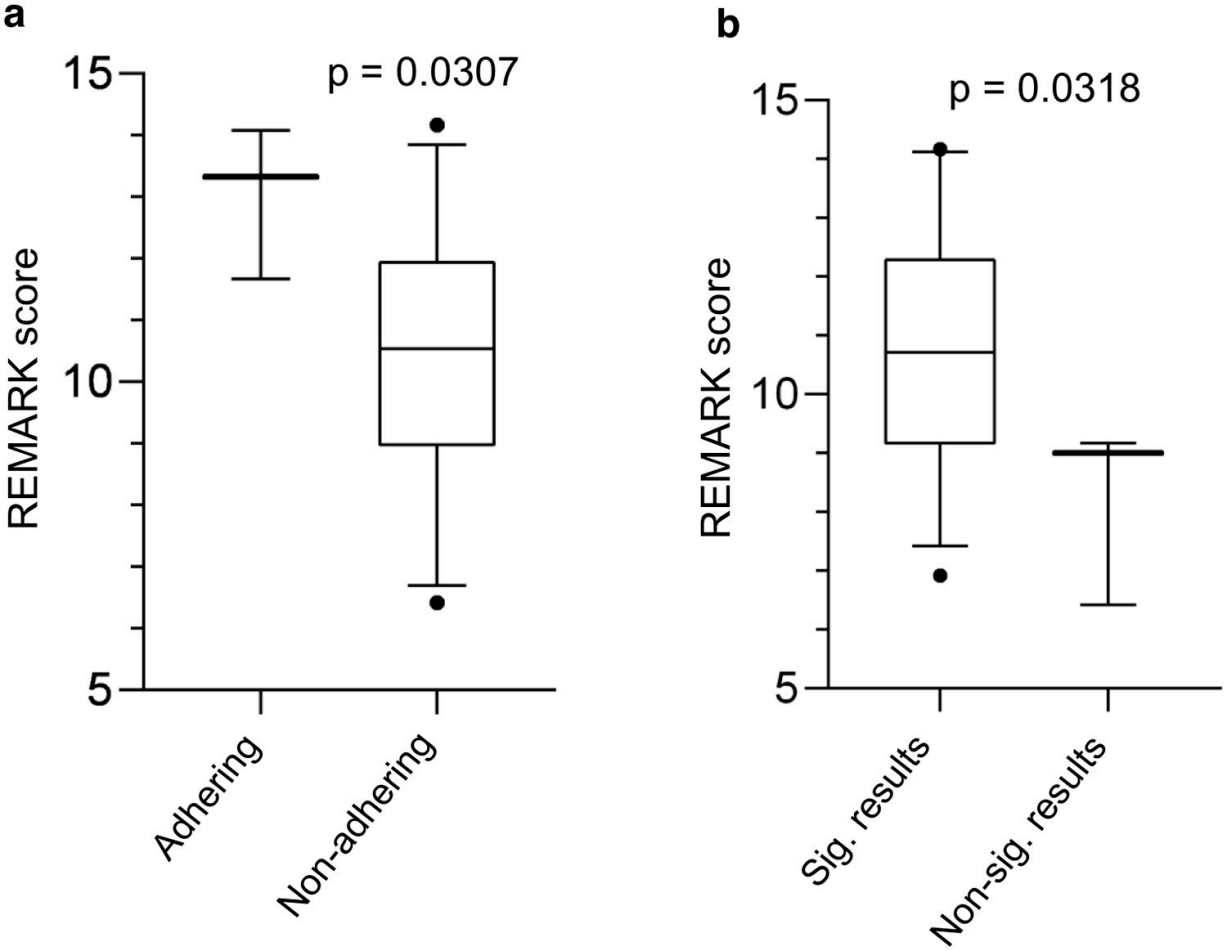
Analysis of categorical variables with REMARK score. Statistical significance was achieved when comparing REMARK scores with: (a) valid manuscripts stating adherence to REMARK guidelines within the body of the paper; and (b) valid manuscripts reporting statistically significant results for their biomarker undergoing evaluation.

**Fig 4:**
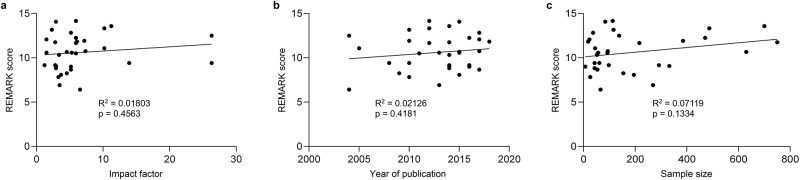
Analysis of continuous variables with REMARK score. Statistical significance was not achieved when analyzing REMARK scores from valid manuscripts and their association with: (a) impact factor; (b) publication year; and (c) sample size.

As shown in Fig 3a and 3b, only papers with a statement of adherence to REMARK guidelines (p = 0.0307) and those reporting statistically significant results (p = 0.0318), respectively, had a statistically significant association with REMARK score. On further analysis of the papers reporting a significant result, there was no difference in REMARK scores of papers reporting only univariate versus only multivariate versus both univariate and multivariate statistically significant results (p = 0.1938). There was no difference in REMARK scores in papers either reporting statistical significance of 1 survival metric versus papers reporting > 1 survival metric (p = 0.2134) or with the type of survival metric reported (p = 0.102). Table 2 lists the remaining categorical variables that failed to reach significance. Fig 4 graphically displays the non-statistically significant results of the continuous variable analysis for impact factor (p = 0.4563), publication year (p = 0.4181) and sample size (p = 0.1334).

## Discussion

We report for the first time that translational studies analyzing RCC circulating prognostic biomarkers need to improve the quality of their methodology and reporting of results. Biomarker development is a challenging area of medical science that requires both step-wise methodological design [25, 26] and transparency in the reporting of methods and results [26–28] to ensure high quality, reproducible and robust conclusions [29–31]. Poor quality reporting of biomarker studies impedes progress and prevents secondary analysis via systematic reviews [25, 32, 33]. Although these issues were the impetus behind the design and publication of the REMARK guidelines, unfortunately they have not been widely or routinely adopted by investigators. A recent study scored prognostic publications of various malignancies in the post-REMARK era using only part of the REMARK criteria and concluded there was a lack of significant improvement in research methodology and/or reporting quality by translational researchers developing prognostic biomarkers [34]. We performed a more in-depth analysis using 48 sub-criteria of the REMARK recommendations. Although our search was restricted to only RCC circulating prognostic biomarker publications, we corroborated prior findings that a paucity of effective study design and reporting persisted. Indeed, a retrospective analysis of prognostic biomarker studies of numerous malignancies published in the pre-REMARK era [35] had similar REMARK scores and sample sizes to those identified in this study.

We explored several correlations with REMARK scores to explain possible causes underlying the paucity of good quality publications. We were only able to show that manuscripts acknowledging adherence to REMARK guidelines and those reporting statistically significant results had higher REMARK scores. However, this observation must be interpreted with caution given the few papers formally reporting adherence to REMARK guidelines (n = 3) or reporting non-significant results (n = 3). Although publications stating adherence to REMARK guidelines did score significantly better relative to publications not citing REMARK, these publications still only achieved a mean score of 13.03 (65%). Furthermore, the top scoring publication from our review did not formally state it was following REMARK guidelines. Our finding that publications stating adherence to REMARK guidelines are of improved quality is in contrast to the 2017 analysis of multiple tumor types and both circulating and non-circulating biomarkers [34]. This difference may be secondary to a more detailed REMARK explanation being published in 2012 [15], shortly before the time their literature search was conducted. This is somewhat in keeping with our search showing papers stating adherence to REMARK were published in 2012 (n = 2) and 2015 (n = 1).

Medical journals with higher impact factors were not associated with better quality publications. This is consistent with a review that also determined poor REMARK scoring in higher impact journals [35], which is also in keeping with poor reporting in non-oncology prognostic studies [36–39]. However, our recording of impact factors was collected at a single time point and not retroactively appraised to the year the study was published, possibly skewing our continuous variable analysis. Our search identified studies from a variety of journals; the limited number of publications per journal prevents intra-journal comparison of study design and reporting quality.

Encouragingly, our study identified a large proportion of publications reporting hazard ratios. This is in contrast to the neuroblastoma prognostic biomarker systematic review that showed approximately 10% of publications reported either a hazard ratio or log_e_(hazard ratio) [40]. However, hazard ratios are dependent upon observed events that are directly related to sample size. Our study showed that > 50% of the manuscripts subjected to REMARK scoring had sample sizes < 100 RCC patients, which is similar to other prognostic papers utilizing small sample sizes [32, 41] that are insufficient to comment on either detection or substantiation of the biomarker being investigated [25, 42–44]. Our study showed very few papers calculated the sample size or effect size. Further, additional bias is introduced by the ongoing concern that specimen availability in pathology laboratory archives are influenced by local referral patterns and/or differences in diagnostic or investigative practices that alter requirement for biopsies or sample collection, thus creating selection bias and limiting the generalizability of the results [45]. A complete description of inclusion/exclusion criteria for sample collection, how the number of included patients was chosen and the methods of collection are necessary to determine the applicability of the results.

This project has several limitations. Our subdivision of each REMARK point is based on the more elaborate REMARK publication [15], but to ensure project feasibility we needed to limit the level of detail. For example, we avoided criteria that were unlikely to be reported, such as: percentage viable cells, specimen adequacy or other details outlined in the Biospecimen Reporting for Improved Study Quality guidelines [46] that is used to appraise the quality of the biological material used in prognostic publications; scoring of DNA- or RNA-quantification assays, antigen retrieval steps or scoring protocols for immunohistochemical assays; or if cause of death was from death certificate or registry. Omission of scoring details may skew our scores, but given the low frequency of publications that satisfied the more generalized criteria, it seems unlikely that publications would be adherent to more granular criteria. Further, our yield of 33 relevant publications is small, which limits our capacity to draw robust and broadly applicable results. Finally, the cost of designing and executing circulating prognostic biomarker studies is not fully captured within REMARK guidelines, which can intentionally result in a lower REMARK score. Note, this systematic review was not registered online.

## Conclusions

Although prognostic biomarkers are valuable tools to aid in clinical decision making, their development is inhibited by poor study design and incomplete reporting. Unfortunately our review shows RCC prognostic circulating biomarker investigations are fraught with similar limitations, possibly explaining their paucity of clinical validity. While publications stating adherence to REMARK guidelines and those reporting statistically significant results appear to be of better quality, they still fail to follow a large proportion of the recommendations. While good reporting will not compensate for poor study design or an ineffective biomarker, it will allow more rapid identification of problematic studies. We suggest that future efforts in RCC prognostic circulating biomarker design should consider the REMARK criteria when designing studies and reporting results to ensure their investigations are high quality, robust and reliable.

## Supporting information

S1 File PRISMA checklist. A list of all PRISMA items and location within the manuscript.
(DOC)

S2 File Search Strategy and MeSH terms. A summary of the terms used in the search, the rationale behind their choice, and associated MeSH terms.
(DOCX)

S3 File Non-suitable publications. A list of all publications not examining RCC circulating prognostic biomarkers and their sub-classifications.
(DOCX)

S1 Table All publications analyzing circulating RCC prognostic biomarkers. A summary list of all abstracts, review papers and primary research publications analyzing circulating RCC prognostic biomarkers. The REMARK score for primary research publications is stated.
(DOCX)

S2 Table REMARK sub-criteria points and correlative variables obtained from each manuscript analyzing circulating RCC prognostic biomarkers. A list of all valid manuscripts with their corresponding REMARK sub-criteria points obtained and correlative variables used in the statistical analysis.
(XLSX)

## Abbreviations

OS: Overall Survival
PFS: Progression Free Survival
PRISMA: Preferred Reporting Items for Systematic Review and Meta-analysis
RCC: renal cell carcinoma
REMARK: Reporting Recommendations for Tumor Marker Prognostic Studies
VEGF: Vascular Endothelial Growth Factor
