# Supplementary material for: Systematic review and REMARK scoring of renal cell carcinoma prognostic circulating biomarker manuscripts
Source: PLoS One. 2019 Oct 22;14(10):e0222359. doi: 10.1371/journal.pone.0222359 (PMC6804962; doi:10.1371/journal.pone.0222359)
Supplement: S2 File — A summary of the terms used in the search, the rationale behind their choice, and associated MeSH terms. (DOCX) [file pone.0222359.s002.docx]

The following terms were used to search PubMed (March 23, 2018), Medline (March 29, 2018) and Embase (March 29, 2018) without any limit on date of past publications: “(renal cell carcinoma OR renal cancer OR kidney cancer OR kidney carcinoma) AND circulating AND (biomarkers OR cell free DNA OR tumor DNA OR methylated cell free DNA OR methylated tumor DNA).”

The specific search terms were chosen to ensure that any variation of renal cell carcinoma would be captured with a specific focus on circulating biomarkers. We emphasized several new circulating biomarkers undergoing active investigation in other disease sites (e.g. tumor DNA, methylated DNA, etc.) to make the study more relevant to the latest biomarker developments.

MeSH terms included:

**Renal cell carcinoma**

- Carcinomas, Renal Cell
- Renal Cell Carcinomas
- Adenocarcinoma, Renal Cell
- Adenocarcinomas, Renal Cell
- Renal Cell Adenocarcinoma
- Renal Cell Adenocarcinomas
- Adenocarcinoma Of Kidney
- Adenocarcinoma Of Kidneys
- Kidney, Adenocarcinoma Of
- Kidneys, Adenocarcinoma Of
- Renal Cell Cancer
- Cancer, Renal Cell
- Cancers, Renal Cell
- Renal Cell Cancers
- Adenocarcinoma, Renal
- Adenocarcinomas, Renal
- Renal Adenocarcinoma
- Renal Adenocarcinomas
- Nephroid Carcinoma
- Carcinoma, Nephroid
- Carcinomas, Nephroid
- Nephroid Carcinomas
- Renal Cell Carcinoma
- Chromophobe Renal Cell Carcinoma
- Sarcomatoid Renal Cell Carcinoma
- Papillary Renal Cell Carcinoma
- Renal Cell Carcinoma, Papillary
- Chromophil Renal Cell Carcinoma
- Clear Cell Renal Cell Carcinoma
- Grawitz Tumor
- Tumor, Grawitz
- Clear Cell Renal Carcinoma
- Carcinoma, Hypernephroid
- Carcinomas, Hypernephroid
- Hypernephroid Carcinoma
- Hypernephroid Carcinomas
- Hypernephroma
- Hypernephromas
- Collecting Duct Carcinoma (Kidney)
- Carcinoma, Collecting Duct (Kidney)
- Carcinomas, Collecting Duct (Kidney)
- Collecting Duct Carcinomas (Kidney)
- Collecting Duct Carcinoma of the Kidney
- Renal Collecting Duct Carcinoma
- Collecting Duct Carcinoma
- Carcinoma, Collecting Duct
- Carcinomas, Collecting Duct
- Collecting Duct Carcinomas

**Renal cancer**

- Kidney Neoplasm
- Neoplasm, Kidney
- Renal Neoplasms
- Neoplasm, Renal
- Neoplasms, Renal
- Renal Neoplasm
- Neoplasms, Kidney
- Cancer of Kidney
- Kidney Cancers
- Renal Cancer
- Cancer, Renal
- Cancers, Renal
- Renal Cancers
- Cancer of the Kidney
- Kidney Cancer
- Cancer, Kidney
- Cancers, Kidney

**Kidney cancer**

- Kidney Neoplasm
- Neoplasm, Kidney
- Renal Neoplasms
- Neoplasm, Renal
- Neoplasms, Renal
- Renal Neoplasm
- Neoplasms, Kidney
- Cancer of Kidney
- Kidney Cancers
- Renal Cancer
- Cancer, Renal
- Cancers, Renal
- Renal Cancers
- Cancer of the Kidney
- Kidney Cancer
- Cancer, Kidney
- Cancers, Kidney

**Kidney carcinoma**

- Carcinomas, Renal Cell
- Renal Cell Carcinomas
- Adenocarcinoma, Renal Cell
- Adenocarcinomas, Renal Cell
- Renal Cell Adenocarcinoma
- Renal Cell Adenocarcinomas
- Adenocarcinoma Of Kidney
- Adenocarcinoma Of Kidneys
- Kidney, Adenocarcinoma Of
- Kidneys, Adenocarcinoma Of
- Renal Cell Cancer
- Cancer, Renal Cell
- Cancers, Renal Cell
- Renal Cell Cancers
- Adenocarcinoma, Renal
- Adenocarcinomas, Renal
- Renal Adenocarcinoma
- Renal Adenocarcinomas
- Nephroid Carcinoma
- Carcinoma, Nephroid
- Carcinomas, Nephroid
- Nephroid Carcinomas
- Renal Cell Carcinoma
- Chromophobe Renal Cell Carcinoma
- Sarcomatoid Renal Cell Carcinoma
- Papillary Renal Cell Carcinoma
- Renal Cell Carcinoma, Papillary
- Chromophil Renal Cell Carcinoma
- Clear Cell Renal Cell Carcinoma
- Grawitz Tumor
- Tumor, Grawitz
- Clear Cell Renal Carcinoma
- Carcinoma, Hypernephroid
- Carcinomas, Hypernephroid
- Hypernephroid Carcinoma
- Hypernephroid Carcinomas
- Hypernephroma
- Hypernephromas
- Collecting Duct Carcinoma (Kidney)
- Carcinoma, Collecting Duct (Kidney)
- Carcinomas, Collecting Duct (Kidney)
- Collecting Duct Carcinomas (Kidney)
- Collecting Duct Carcinoma of the Kidney
- Renal Collecting Duct Carcinoma
- Collecting Duct Carcinoma
- Carcinoma, Collecting Duct
- Carcinomas, Collecting Duct
- Collecting Duct Carcinomas

**Circulating**

- Neoplasm Circulating Cells
- Circulating Neoplastic Cells
- Cell, Circulating Neoplastic
- Cells, Circulating Neoplastic
- Circulating Neoplastic Cell
- Neoplastic Cell, Circulating
- Circulating Tumor Cells
- Cell, Circulating Tumor
- Cells, Circulating Tumor
- Circulating Tumor Cell
- Tumor Cell, Circulating
- Tumor Cells, Circulating
- Cells, Neoplasm Circulating
- Cell, Neoplasm Circulating
- Neoplasm Circulating Cell
- Circulating Cells, Neoplasm
- Tumor Cells, Embolic
- Cell, Embolic Tumor
- Cells, Embolic Tumor
- Embolic Tumor Cell
- Tumor Cell, Embolic
- Embolic Tumor Cells
- Embolism, Tumor
- Embolisms, Tumor
- Tumor Embolism
- DNA, Circulating Tumor
- Tumor DNA, Circulating
- Cell-Free Tumor DNA
- Cell Free Tumor DNA
- DNA, Cell-Free Tumor
- Tumor DNA, Cell-Free
- MicroRNA, Circulating
- Cell-Free MicroRNA
- Cell Free MicroRNA
- MicroRNA, Cell-Free
- Cell Free Nucleic Acids
- Nucleic Acids, Cell-Free
- Circulating Cell-Free Nucleic Acids
- Circulating Cell Free Nucleic Acids
- Circulating Nucleic Acids
- Acids, Circulating Nucleic
- Nucleic Acids, Circulating
- Cell-Free Nucleic Acid
- Cell Free Nucleic Acid
- Nucleic Acid, Cell-Free
- Cell-Free DNA
- Cell Free DNA
- DNA, Cell-Free
- cfDNA
- cirDNA
- Cell-Free Deoxyribonucleic Acid
- Acid, Cell-Free Deoxyribonucleic
- Cell Free Deoxyribonucleic Acid
- Deoxyribonucleic Acid, Cell-Free
- Circulating DNA
- DNA, Circulating
- Cell-Free RNA
- Cell Free RNA
- RNA, Cell-Free
- cfRNA
- cirRNA
- Cell-Free Ribonucleic Acid
- Acid, Cell-Free Ribonucleic
- Cell Free Ribonucleic Acid
- Ribonucleic Acid, Cell-Free
- Circulating RNA
- RNA, Circulating
- Biopsies, Liquid
- Biopsy, Liquid
- Liquid Biopsies

**Biomarkers**

- Markers, Biological
- Biologic Markers
- Markers, Biologic
- Biologic Marker
- Marker, Biologic
- Marker, Biological
- Biological Marker
- Biological Markers
- Markers, Laboratory
- Laboratory Markers
- Laboratory Marker
- Marker, Laboratory
- Serum Markers
- Markers, Serum
- Marker, Serum
- Serum Marker
- Surrogate Endpoints
- Endpoints, Surrogate
- Surrogate End Points
- End Points, Surrogate
- Surrogate End Point
- End Point, Surrogate
- Surrogate Endpoint
- Endpoint, Surrogate
- Markers, Clinical
- Clinical Markers
- Clinical Marker
- Marker, Clinical
- Viral Markers
- Markers, Viral
- Viral Marker
- Marker, Viral
- Biochemical Marker
- Biochemical Markers
- Markers, Biochemical
- Marker, Biochemical
- Markers, Immunologic
- Immune Markers
- Markers, Immune
- Marker, Immunologic
- Immunologic Markers
- Immune Marker
- Marker, Immune
- Immunologic Marker
- Surrogate Markers
- Markers, Surrogate
- Marker, Surrogate
- Tumor Biomarkers
- Markers, Biological Tumor
- Tumor Markers, Biological
- Markers, Tumor Metabolite
- Tumor Metabolite Markers
- Metabolite Markers, Tumor
- Marker, Tumor Metabolite
- Metabolite Marker, Tumor
- Tumor Metabolite Marker
- Tumor Markers, Biologic
- Biologic Tumor Markers
- Markers, Biologic Tumor
- Marker, Biologic Tumor
- Biologic Tumor Marker
- Tumor Marker, Biologic
- Biochemical Tumor Markers
- Markers, Biochemical Tumor
- Marker, Biochemical Tumor
- Biochemical Tumor Marker
- Tumor Marker, Biochemical
- Tumor Markers, Biochemical
- Carcinogen Markers
- Markers, Carcinogen
- Markers, Neoplasm Metabolite
- Neoplasm Metabolite Markers
- Marker, Neoplasm Metabolite
- Metabolite Marker, Neoplasm
- Neoplasm Metabolite Marker
- Metabolite Markers, Neoplasm
- Biological Tumor Markers
- Biological Tumor Marker
- Tumor Marker, Biological
- Marker, Biological Tumor
- Markers, Tumor
- Tumor Markers
- Biomarkers, Cancer
- Cancer Biomarkers

**Cell free DNA**

- Cell Free Nucleic Acids
- Nucleic Acids, Cell-Free
- Circulating Cell-Free Nucleic Acids
- Circulating Cell Free Nucleic Acids
- Circulating Nucleic Acids
- Acids, Circulating Nucleic
- Nucleic Acids, Circulating
- Cell-Free Nucleic Acid
- Cell Free Nucleic Acid
- Nucleic Acid, Cell-Free
- Cell-Free DNA
- Cell Free DNA
- DNA, Cell-Free
- cfDNA
- cirDNA
- Cell-Free Deoxyribonucleic Acid
- Acid, Cell-Free Deoxyribonucleic
- Cell Free Deoxyribonucleic Acid
- Deoxyribonucleic Acid, Cell-Free
- Circulating DNA
- DNA, Circulating
- Cell-Free RNA
- Cell Free RNA
- RNA, Cell-Free
- cfRNA
- cirRNA
- Cell-Free Ribonucleic Acid
- Acid, Cell-Free Ribonucleic
- Cell Free Ribonucleic Acid
- Ribonucleic Acid, Cell-Free
- Circulating RNA
- RNA, Circulating

**Tumor DNA**

- DNA, Circulating Tumor
- Tumor DNA, Circulating
- Cell-Free Tumor DNA
- Cell Free Tumor DNA
- DNA, Cell-Free Tumor
- Tumor DNA, Cell-Free

**Methylated cell free DNA**

- No MeSH terms listed

**Methylated tumor DNA**

- No MeSH terms listed
