# Supplementary material for: Systematic review and REMARK scoring of renal cell carcinoma prognostic circulating biomarker manuscripts
Source: PLoS One. 2019 Oct 22;14(10):e0222359. doi: 10.1371/journal.pone.0222359 (PMC6804962; doi:10.1371/journal.pone.0222359)
Supplement: S3 File — A list of all publications not examining RCC circulating prognostic biomarkers and their sub-classifications. (DOCX) [file pone.0222359.s003.docx]

Non-suitable publications = 438

♦ Air pollution = 1

♦ Aging = 1

♦ Acute/chronic kidney disease = 11

♦ Amyloidosis = 1

♦ Bioenergetics and metabolism = 2

♦ Biomarker(s) for > 1 disease (not including RCC) = 20

♦ Breast cancer = 3

♦ Case report^a^ = 25

♦ Calcium homeostasis = 4

♦ Cardiovascular = 5

♦ Cytomegalovirus = 1

♦ Colorectal cancer = 4

♦ Commentary letter = 11

♦ Conference proceeding introduction = 2

♦ Cushing’s disease = 1

♦ Diabetes = 2

♦ Endocrine diseases (multiple conditions) = 1

♦ Endometrial cancer = 1

♦ Gastric cancer = 1

♦ Hematology (malignant) = 5

♦ Hepatitis = 1

♦ HIF-1α pathway = 1

♦ HIV = 1

♦ Limb ischemia = 1

♦ Liver cancer = 3

♦ Lung cancer = 3

♦ Melanoma = 6

♦ Membranous nephropathy = 1

♦ Methodology and instrumentation = 50

♦ Nasopharyngeal cancer = 1

♦ Neuroendocrine tumors = 3

♦ Neurofibromatosis = 1

♦ Non-RCC renal cancer biomarkers = 2

♦ No diagnostic, predictive or prognostic biomarkers = 129

♦ Obesity = 2

♦ Oncolytic virus therapy = 1

♦ Ovarian cancer = 1

♦ Pancreatic cancer = 2

♦ Prostate cancer = 5

♦ Proximal tubular secretion = 1

♦ Pulmonary fibrosis = 1

♦ RCC diagnostic circulating biomarkers = 46^b^

♦ RCC predictive circulating biomarkers = 33

♦ RCC tumor biomarkers = 13

♦ RCC urinary biomarkers = 6

♦ Sarcoma = 1

♦ Sepsis = 1

♦ Trauma = 1

♦ Thyroid cancer = 2

♦ Transitional cell carcinoma = 3

♦ Trials in progress or to be performed = 10

♦ Transplantation = 3

♦ Vascular damage = 1

^a^ Papers are classified in this section regardless if they are reporting on biomarkers or RCC.

^b^ In total, 5 publications analyzing RCC prognostic circulating biomarkers also analyzed RCC diagnostic circulating biomarkers. These 5 studies were tallied in the “Publications examining RCC circulating prognostic biomarkers” section in **Fig 1**.
