# Supplementary material for: Systematic review and REMARK scoring of renal cell carcinoma prognostic circulating biomarker manuscripts
Source: PLoS One. 2019 Oct 22;14(10):e0222359. doi: 10.1371/journal.pone.0222359 (PMC6804962; doi:10.1371/journal.pone.0222359)
Supplement: S1 Table — A summary list of all abstracts, review papers and primary research publications analyzing circulating RCC prognostic biomarkers. The REMARK score for primary research publications is stated. (DOCX) [file pone.0222359.s004.docx]

| **SUPPORTING TABLE 1: All publications analyzing circulating RCC prognostic biomarkers** | | | |
| --- | --- | --- | --- |
| **Type of publication** | **Subjected to REMARK analysis** | **Reference** | **REMARK score** |
| **Cell free DNA** | | | |
| Review | No | Expert Rev Mol Med. 2018 Jan 18;20:e1. | N/A |
| Manuscript | Yes | Oncol Res Treat. 2017;40(11):707-710. | 12.08 |
| Manuscript | Yes | Urol Int. 2013;91(3):273-8. | 10.58 |
| Abstract | No | European Urology, Supplements. Conference: 26th Annual Congress of the European Association of Urology, EAU. Vienna Austria. Conference Publication: (var.pagings). 10 (2) (pp 105), 2011. Date of Publication: March 2011. | N/A |
| **Endothelial cells** | | | |
| Manuscript | Yes | BMC Cancer. 2010 Dec 31;10:695. | 7.83 |
| **Hematopoietic cells** | | | |
| Manuscript | Yes | Ann Oncol. 2011 Apr;22(4):815-20. | 9.42 |
| Review | No | Pharmacogenomics. 2006 Mar;7(2):187-202. | N/A |
| Manuscript | Yes | Clin Cancer Res. 2005 Feb 1;11(3):1181-9. | 11.08 |
| **Metalloproteinases** | | | |
| Manuscript | Yes | Urol Oncol. 2014 Jul;32(5):584-8. | 10.33 |
| **microRNA** | | | |
| Manuscript | Yes | Clin Epigenetics. 2018 Jan 23;10:11. | 11.83 |
| Manuscript | Yes | Oncotarget. 2017 Oct 11;8(61):103315-103326. | 8.67 |
| Manuscript | Yes | Int J Cancer. 2017 Nov 1;141(9):1730-1740. | 10.75 |
| Review | No | Int J Biol Markers. 2016 Feb 28;31(1):e26-37. | N/A |
| Manuscript | Yes | Int J Mol Sci. 2015 Sep 29;16(10):23382-9. | 8.08 |
| Review | No | Biomark Res. 2014 Oct 22;2:19. | N/A |
| Review | No | Front Oncol. 2014 Mar 17;4:49. | N/A |
| Manuscript | Yes | Tumour Biol. 2014 May;35(5):4057-66. | 8.83 |
| Review | No | J Kidney Cancer VHL. 2014 Dec 24;1(8):84-98. | N/A |
| Abstract | No | European Journal of Cancer. Conference: European Cancer Congress 2015, ECC 2015. Vienna Austria. Conference Publication: (var.pagings). 51 (SUPPL. 3) (pp S36-S37), 2015. Date of Publication: September 2015. | N/A |
| Abstract | No | Journal of Clinical Oncology. Conference: 2012 Annual Meeting of the American Society of Clinical Oncology, ASCO. Chicago, IL United States. Conference Publication: (var.pagings). 30 (15 SUPPL. 1) (no pagination), 2012. Date of Publication: 20 May 2012. | N/A |
| **Tumor cells** | | | |
| Review | No | Ther Adv Urol. 2017 Nov 22;10(2):65-77. | N/A |
| Review | No | Nat Rev Urol. 2017 Feb;14(2):90-97. | N/A |
| Manuscript | Yes | Cancer Epidemiol Biomarkers Prev. 2009 Aug;18(8):2190-4. | 8.25 |
| Manuscript | No, paper not accessible | Oncol Rep. 2005 Oct;14(4):895-9. | N/A |
| **Vascular endothelial growth factor and pro-angiogenic cytokines** | | | |
| Manuscript | Yes | Clin Cancer Res. 2013 Feb 15;19(4):929-37. | 13.33 |
| Manuscript | Yes | Oncotarget. 2017 Jun 27;8(26):42149-42158. | 12.83 |
| Review | No | Urol Clin North Am. 2016 Feb;43(1):95-104. | N/A |
| Review | No | Clin Cancer Res. 2014 Apr 15;20(8):2060-71. | N/A |
| Manuscript | Yes | Cancer Chemother Pharmacol. 2014 Jan;73(1):151-61. | 11.75 |
| Manuscript | Yes | J Clin Oncol. 2008 Aug 1;26(22):3743-8. | 9.42 |
| Review | No | J Clin Oncol. 2013 Mar 20;31(9):1219-30. | N/A |
| Abstract | No | Journal of Clinical Oncology. Conference: ASCO Annual Meeting 2011. Chicago, IL United States. Conference Publication: (var.pagings). 29 (15 SUPPL. 1) (no pagination), 2011. Date of Publication: 20 May 2011. | N/A |
| Review | No | Nat Rev Clin Oncol. 2009 Jun;6(6):327-38. | N/A |
| Review | No | Cancer J. 2011 Mar-Apr;17(2):134-41. | N/A |
| **Vitamin levels** | | | |
| Manuscript | Yes | Cancer Epidemiol Biomarkers Prev. 2015 Aug;24(8):1277-81. | 10.67 |
| Manuscript | Yes | J Natl Cancer Inst. 2014 Nov 5;106(12). | 13.58 |
| **Categorized in ≥ 2 sections** | | | |
| Review | No | Curr Oncol Rep. 2012 Jun;14(3):221-9. | N/A |
| Review | No | J Cancer. 2015 Sep 5;6(11):1105-13. | N/A |
| Review | No | Crit Rev Clin Lab Sci. 2014 Aug;51(4):200-31. | N/A |
| Review | No | Cancer Sci. 2011 Nov;102(11):1949-57. | N/A |
| Review | No | Urol Oncol. 2016 Nov;34(11):510-518. | N/A |
| Manuscript | Yes | Oncotarget. 2016 Apr 12;7(15):20109-23. | 9.00 |
| Manuscript | Yes | Cancer Chemother Pharmacol. 2014 Oct;74(4):739-50. | 9.17 |
| Manuscript | Yes | Clin Chim Acta. 2016 Jan 15;452:109-19. | 14.08 |
| Review | No | Cancer. 2009 May 15;115(10 Suppl):2346-54. | N/A |
| Review | No | Curr Opin Urol. 2003 Nov;13(6):457-62. | N/A |
| Review | No | Urol Oncol. 2000 Jul 1;5(4):139-148. | N/A |
| Manuscript | Yes | Oncologist. 2015 Oct;20(10):1140-8. | 10.58 |
| Abstract | No | Annals of Oncology. Conference: 42nd ESMO Congress, ESMO 2017. Spain. 28 (Supplement 5) (pp v40), 2017. Date of Publication: September 2017. | N/A |
| Abstract | No | Annals of Oncology. Conference: 19th National Congress of Medical Oncology. Italy. 28 (Supplement 6) (pp vi18), 2017. Date of Publication: October 2017. | N/A |
| Review | No | Clin Transl Oncol. 2016 Jan;18(1):1-8. | N/A |
| Abstract | No | European Journal of Cancer. Conference: European Cancer Congress 2015, ECC 2015. Vienna Austria. Conference Publication: (var.pagings). 51 (SUPPL. 3) (pp S34), 2015. Date of Publication: September 2015. | N/A |
| Abstract | No | Journal of Clinical Oncology. Conference: 2014 Genitourinary Cancers Symposium. San Francisco, CA United States. Conference Publication: (var.pagings). 32 (4 SUPPL. 1) (no pagination), 2014. Date of Publication: 01 Feb 2014. | N/A |
| Review | No | Acta Clin Belg. 2011 Sep-Oct;66(5):332-6. | N/A |
| Abstract | No | Journal of Clinical Oncology. Conference: 2009 Annual Meeting of the American Society of Clinical Oncology, ASCO. Orlando, FL United States. Conference Publication: (var.pagings). 27 (15 SUPPL. 1) (pp 5114), 2009. Date of Publication: 20 May 2009. | N/A |
| Manuscript | Yes | Br J Cancer. 2011 Mar 29;104(7):1144-50. | 10.50 |
| Manuscript | Yes | Br J Cancer. 2012 Sep 25;107(7):1131-7. | 11.67 |
| Manuscript | Yes | Br J Cancer. 2016 Mar 15;114(6):642-9. | 12.25 |
| Review | No | Semin Oncol. 2013 Aug;40(4):459-64. | N/A |
| Manuscript | Yes | J Clin Oncol. 2004 Jun 15;22(12):2371-8. | 12.50 |
| Abstract | No | European Journal of Cancer. Conference: 6th Asian Oncology Summit and 10th Annual Conference of the Organisation for Oncology and Translational Research. Kuala Lumpur Malaysia. Conference Publication: (var.pagings). 50 (SUPPL. 4) (pp e65), 2014. Date of Publication: May 2014. | N/A |
| Abstract | No | Annals of Oncology. Conference: 37th ESMO Congress. Vienna Austria. Conference Publication: (var.pagings). 23 (SUPPL. 9) (pp ix261), 2012. Date of Publication: September 2012. | N/A |
| Abstract | No | BJU International. Conference: 13th International Kidney Cancer Symposium. Chicago, IL United States. Conference Publication: (var.pagings). 114 (SUPPL. 4) (pp 17), 2014. Date of Publication: October 2014. | N/A |
| **Other** | | | |
| Manuscript | Yes | J Mol Biomark Diagn. 2016 Jun;1(Suppl 2). | 9.17 |
| Manuscript | Yes | Dis Markers. 2014;2014:689795. | 9.08 |
| Manuscript | Yes | Br J Cancer. 2012 Feb 28;106(5):904-8. | 14.17 |
| Manuscript | Yes | Urology. 2010 Aug;76(2):513.e1-6. | 13.17 |
| Manuscript | Yes | Cancer. 2004 Sep 1;101(5):963-8. | 6.42 |
| Abstract | No | Cancer Research. Conference: 107th Annual Meeting of the American Association for Cancer Research, AACR 2016. United States. 76 (14 Supplement) (no pagination), 2016. Date of Publication: 2016. | N/A |
| Review | No | Cell Stress Chaperones. 2005 Summer;10(2):86-103. | N/A |
| Review | No | Nat Rev Cancer. 2014 May;14(5):329-41. | N/A |
| Manuscript | Yes | Eur J Cancer. 2010 Jul;46(10):1927-35. | 11.92 |
| Manuscript | Yes | Acta Oncol. 2013 Jan;52(1):159-65. | 6.92 |
